# On the Role of Training Data for SVM-Based Microwave Brain Stroke Detection and Classification

**DOI:** 10.3390/s23042031

**Published:** 2023-02-10

**Authors:** Tomas Pokorny, Jan Vrba, Ondrej Fiser, David Vrba, Tomas Drizdal, Marek Novak, Luca Tosi, Alessandro Polo, Marco Salucci

**Affiliations:** 1 Department of Biomedical Technology, Faculty of Biomedical Engineering, Czech Technical University in Prague, 166 00 Prague, Czech Republic; 2 ELEDIA Research Center (ELEDIA@UniTN—University of Trento), DICAM—Department of Civil, Environmental, and Mechanical Engineering, Via Mesiano 77, 38123 Trento, Italy

**Keywords:** SVM, brain stroke, microwave devices, numerical model

## Abstract

The aim of this work was to test microwave brain stroke detection and classification using support vector machines (SVMs). We tested how the nature and variability of training data and system parameters impact the achieved classification accuracy. Using experimentally verified numerical models, a large database of synthetic training and test data was created. The models consist of an antenna array surrounding reconfigurable geometrically and dielectrically realistic human head phantoms with virtually inserted strokes of arbitrary size, and different dielectric parameters in different positions. The generated synthetic data sets were used to test four different hypotheses, regarding the appropriate parameters of the training dataset, the appropriate frequency range and the number of frequency points, as well as the level of subject variability to reach the highest SVM classification accuracy. The results indicate that the SVM algorithm is able to detect the presence of the stroke and classify it (i.e., ischemic or hemorrhagic) even when trained with single-frequency data. Moreover, it is shown that data of subjects with smaller strokes appear to be the most suitable for training accurate SVM predictors with high generalization capabilities. Finally, the datasets created for this study are made available to the community for testing and developing their own algorithms.

## 1. Introduction

Microwave (MW) technology enables the development of an affordable, non-invasive, compact, lightweight, and therefore, a portable diagnostic system suitable for pre-hospital care. In contrast to standard medical imaging methods such as computed tomography (CT) and magnetic resonance imaging (MRI), MW stroke diagnostic systems could thus be included in ambulance vehicles. This could reduce the time to stroke classification and thus speed up the initiation of treatment reducing the lasting effects of strokes on patients [1].

MW detection and classification of strokes is based on measuring changes in dielectric properties of the brain during stroke progression [2]. For this purpose, an antenna array is placed around the patient’s head and the reflection and transmission coefficients at the antenna ports are measured. In supervised machine learning approaches [3,4], the algorithm is trained using data for known (“labeled”) scenarios (i.e., the existence of a stroke and, eventually, its type). Training data can be obtained either synthetically using numerical simulations or by measurements of phantoms or human subjects. However, this is almost impossible, as it requires at least data from hundreds of patients who are in life-threatening danger at any given time [5,6].

In [7,8], two brain stroke types are classified using inverse FFT transformation S-matrix. In [9,10], a human head model with intracranial hemorrhage was used to investigate the ability of a machine learning-based classification algorithm to distinguish healthy individuals from subjects with intracranial hemorrhage, depending on the number of subjects for training.

In [11], the first version of a laboratory MW system developed in our group in 2015, a homogeneous head phantom was used in experimental measurements, which demonstrated the potential of MW systems for brain stroke classifications/diagnostics. In [12], the SVM algorithm was used to detect the presence of a stroke on a simplified head phantom. Very high stroke detection accuracy was achieved even with a limited amount of data used for learning the algorithm. A follow-up work [13] dealt with the distinction between ischemic and hemorrhagic cerebrovascular events. From [14,15], where the classification results using different machine learning algorithms (Support Vector Machines, Nearest Neighbors, Discriminant analysis, Naïve Bayes classifier and Classification Tree) were compared, it suggests that SVM might be the most suitable for this application.

The aim of this study was to test the influence of the variability of the training data sets on the detection and classification accuracy using the SVM algorithm. Further, based on these results, we want to be able to recommend suitable properties of the training dataset. For this purpose, several 2D numerical models were created and experimentally verified consisting of an antenna array surrounding reconfigurable geometrically and dielectrically realistic human head models including virtually embedded stroke phantoms. The human head models were based on 10 different real subject heads. Thus, a large database of training and test datasets was created. The SVM-based algorithm was tested for the detection of the stroke presence and the classification of its type. For the SVM-based classification of strokes, we tested the following hypotheses:

**Hypothesis** **1** **(H1).** *The most suitable training data are from subjects with small strokes.*

**Hypothesis** **2** **(H2).** *Single-frequency and multi-frequency training data lead to the same classification accuracy.*

**Hypothesis** **3** **(H3).** *A SVM trained on data from subjects with small strokes can accurately classify randomly sized strokes.*

**Hypothesis** **4** **(H4).** *A SVM trained on data from patients with small strokes can accurately classify randomly sized strokes at random positions.*

## 2. Materials and Methods

### 2.1. Numerical Simulations

In this study, COMSOL Multiphysics [16] was used to create 2D synthetic training and test data in the form of transmission and reflection coefficients (called scattering parameters or, shortly, S-parameters). The corresponding 2D numerical models consist of a fixed antenna array surrounding reconfigurable geometrically and dielectrically realistic human head models. The geometry of the antenna array model corresponds to a cross-section of a laboratory prototype of a microwave imaging system described in [11].

### 2.2. Numerical Model

**Figure 1:**
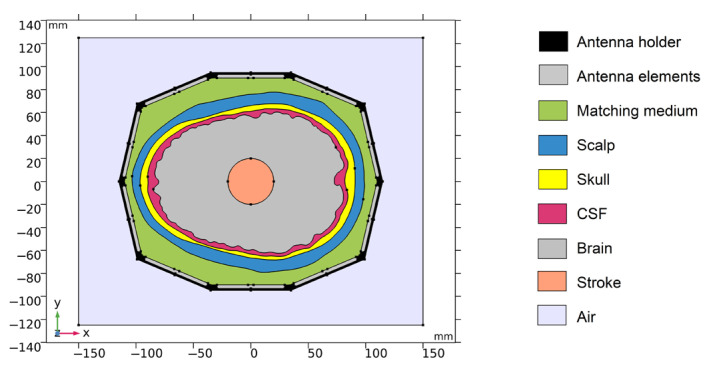
Geometry of the 2D numerical model. Antenna holder and antenna elements surrounding a realistic human head model. XY-plane corresponds to the cross-section at z = 100 mm of the laboratory prototype 3D numerical model.

The geometry of one numerical model is depicted in Figure 1.

**Figure 2:**
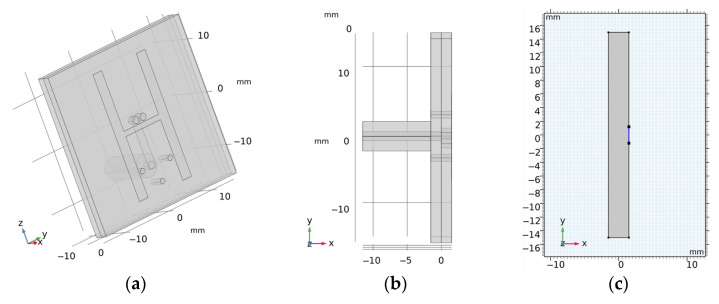
(**a**,**b**) is the 3D geometry of the antennas and (**c**) is the final 2D equivalent antenna model, where segments representing the slots (marked in blue) were changed to achieve maximum agreement to the measured transmission parameters within laboratory system.

The 2D geometry was created from the cross-section of the laboratory prototype 3D numerical model in 100 mm z-coordinates. Absorption boundary conditions are set around the entire outer perimeter of the computational domain. The antenna elements used in this study are inspired by the slot antenna from [17]. An antenna 2D equivalent model consists of a rectangle representing a conductive cavity and a slot was used in simulations (see Figure 2). A slot segment was created on the perimeter of the rectangle facing the displayed area and the boundary condition of the port (“User defined”, Ez) was assigned to it. The rest of the perimeter of the rectangle antenna was assigned a perfect electric conductor boundary condition. The sizes of the antenna segments representing the slots were changed to find the global minimum of a cost function using the genetic algorithm. We minimized the transmission parameter differences between the optimized 2D model and the reference experimental measurement for the entire 10-port system. The inner part of the antenna array was filled only with the matching medium. The maximum possible agreement between the transfer coefficients for 2D numerical simulation and experimental measurement was achieved. Relative magnitude differences were calculated according to Equation (1). Antennas operate in the frequency range from 0.8 GHz to 2 GHz. A triangular mesh was used, where the maximum value of the side length was set to 1/8 of the transverse electromagnetic wavelength in the given environment and for the highest frequency equal to 2 GHz.

**Figure 3:**
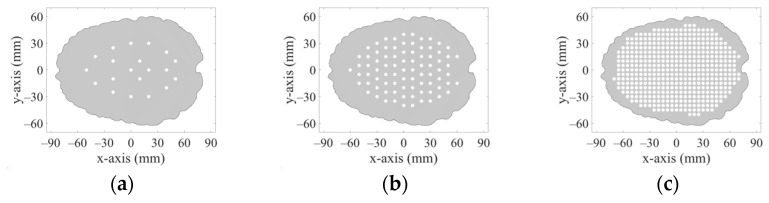
20 positions (**a**), 83 positions (**b**), and 456 positions (**c**) in the brain where strokes of various sizes were placed.

The 2D geometries of the models of all 10 human heads used here are based on 3D head models from the IT’IS Foundation’s “The Population Head Model V1.0” database [18]. In Materialize 3-matic software [19], the mesh of the models was modified so that it could be imported and used for FEM simulations in COMSOL Multiphysics. 2D models were created from a section of 3D models in the brain part of the head. The models contain layers representing the scalp, skull, cerebrospinal fluid, and brain (white matter and grey matter). For completeness, the geometries of the 10 head phantoms are depicted in Appendix A. The stroke locations are represented by a circle with diameters from 20 to 40 mm placed in different positions in the brain domain shown in Figure 3.

### 2.3. Dielectric Properties of Biological Tissues

Realistic dielectric properties values were assigned to the Individual domains of the human head models, which represents different biological tissues. These values were determined using the frequency-dependent 4-pole Cole–Cole model [20]. Specifically, for the skull, CSF, and hemorrhagic stroke represented by the blood, the 4-pole Cole–Cole model parameters were directly adopted from the database [21]. For the brain dielectric properties, we used average values of white matter, gray matter and cerebellum and for the scalp average values of skin and fat. The dielectric properties of the individual domains at 1 GHz are shown in Table 1.

### 2.4. Numerical Models Validation

**Figure 4:**
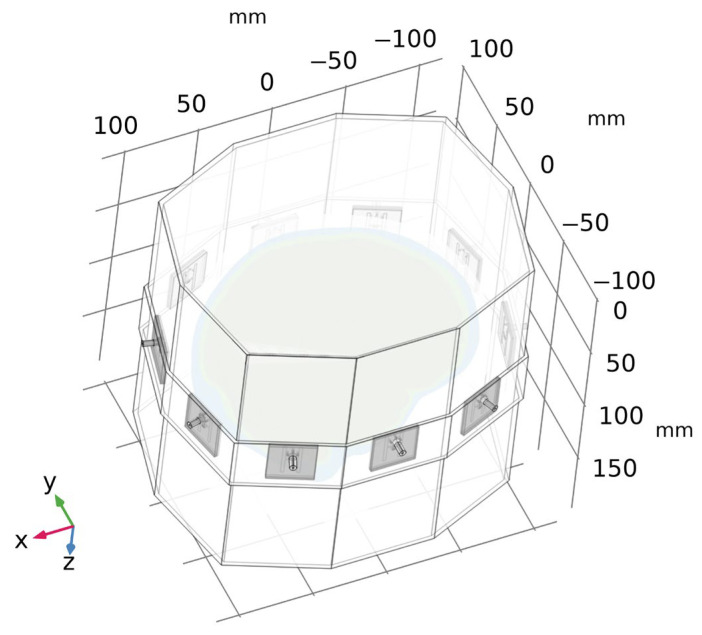
A geometry of a 3D numerical model of the laboratory prototype of microwave imaging system. For clarity, the air surrounding the antenna array is omitted in the figure.

**Figure 5:**
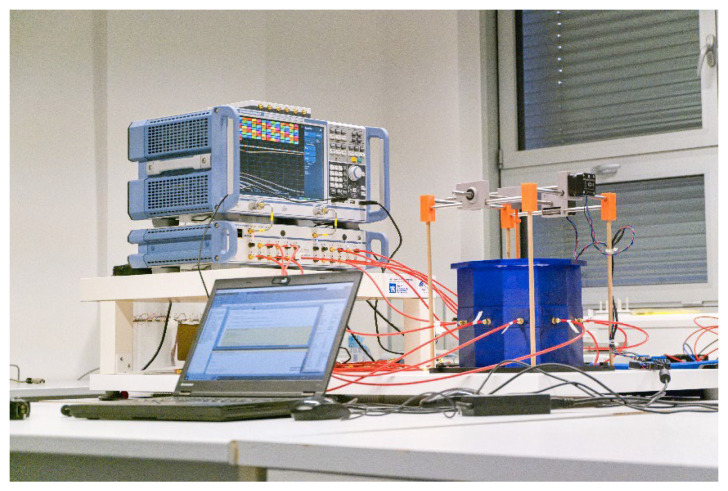
A laboratory prototype of the microwave imaging system [11] consisting of an in-house designed antenna array and a positioning system for stroke models, and commercial measuring devices: a vector network analyzer ZNB8, and a switching matrix ZN-Z84, both Rohde Schwartz, Germany.

Validation of the numerical models was done in two steps. First, we directly compared the (synthetic) S-parameters obtained from simulations using 2D and 3D models (the geometries of these models are shown in Figure 1 and Figure 4) and measurements using the laboratory prototype of a MW imaging system [11] shown in Figure 5.

For the 2D and 3D simulations and measurements, the inner part of the antenna array was filled only with the matching medium, which allowed us idealized comparisons of all these scenarios.

Relative magnitudes differences are defined as:

(1) $$\Delta M_{ij}=20\cdot \log_{10}\left(\left|\;\frac{S_{ij}^{A}}{S_{ij}^{B}}\right|\right),$$

where $S_{ij}^{A}$ and $S_{ij}^{B}$ denote the scattering parameters in the presence or absence of a stroke, respectively, for each *i*, *j* antennas pair.

Magnitudes differences of S-parameters are listed in Appendix C.

### 2.5. Acquisition, Nature, and Variability of Training and Testing Data Sets

The datasets were automatically generated using the reconfigurable numerical model described in Section 2.2 and 2D simulations done in COMSOL Multiphysics controlled with in-house written MATLAB scripts. These scripts specifically set the operating frequency, stroke type, size and position, head phantom size scaling, and saved the resulting S-matrices together with the numerical model settings to a MATLAB structure matrix file.

According to studies [5,13], the most suitable operating frequencies for MW imaging are around 1 GHz. Our datasets contain simulation results for 25 equidistant frequency points ranging from 0.8 to 2 GHz.

The head models were based on 10 different patient-specific geometries. To increase the variability of tested datasets, the geometries of the head models were scaled to 95–105% of their original size independently in the *x* and *y* directions. The scaling factors were determined for each data point using a uniform probability density random number generator. We used 2 types of strokes: Ischemic stroke (iStroke) caused by blockage of a blood vessel by a clot and hemorrhagic stroke (hStroke) caused by intracranial bleeding, also the scenario without stroke (noStroke) was used. Three datasets have been generated as summarized in the text below and Table 2.

DataSet 1 includes 2D simulation results for subjects with five different stroke sizes (20, 25, 30, 35 and 40 mm in diameter) of both stroke types placed at 20 predefined positions within randomly chosen and scaled head models. In total, 1000 simulations were for each stroke type, and 1000 simulations of randomly scaled head models without stroke. Therefore, 3000 calculations of S–matrices were done for one frequency point. DataSet1 was subsequently supplemented with data generated in the same way for 83 and 456 different stroke positions.

DataSet 2 includes the simulations’ results for subjects with ischemic and hemorrhagic strokes of random size (ranging from 20 to 40 mm in diameter determined using uniform probability density random number generator) placed at 20 predefined positions within randomly chosen and scaled head models. In total, 200 simulations were computed for each type of stroke and 200 simulations for randomly scaled head models without stroke.

DataSet 3 includes the simulations’ results for subjects with ischemic and hemorrhagic strokes of random size (range from 20 to 40 mm and determined using uniform probability density random number generator) placed at random positions within randomly chosen and scaled head models. In total, 200 simulations were calculated for each type of stroke and 200 simulations for randomly scaled head models without stroke.

### 2.6. Feature Selection and Extraction

As previously mentioned, the numerical models contain, in total, 10 antenna elements. For each simulation, we thus obtain a complex S-matrix with dimensions of 10 × 10. Thanks to the Lorentz principle of reciprocity $\left(S_{ij}=S_{ji}\right)$ there are only 55 independent elements of the S-matrix. We divided the complex values of these S-parameters into real and imaginary parts and thus obtained 110 observed features which give us 110 dataset dimensions for one frequency point [15].

The training and test data were centered and normalized to a range from 0 to 1. For training data, we calculate the principal component coefficients using Principal Component Analysis (PCA) through Singular Value Decomposition (SVD). The principal component coefficient was used to reduce the dimension for training and test data sets. The most appropriate number of data dimensions (i.e., of PCA-extracted features) was chosen based on the classification accuracy for different dimension sizes.

### 2.7. Stroke Classification

For stroke detection and classification, we used a non-linear SVM with kernel function [22]. The kernel function maps the data to a higher-dimensional space, where the classes are easier to separate. For the classification between head phantom with ischemic stroke (iStroke), hemorrhagic stroke (hStroke), and head phantom without stroke (noStroke) classes, we constructed a multiclass classifier by combining multiple binary classifiers [13]. To improve the performance of the algorithm, we searched for the best settings of the SVM algorithm by optimizing hyper parameters based on Bayesian optimization.

To train the algorithm, an input matrix of a known set of input samples (TrainData) and an input matrix of known responses to the samples (TrainDataClass) were prepared according to the tested hypothesis. The input matrix of known samples consisted of a predefined number of rows in which every row was filled with a single-measured S-matrix, normalized, and dimensionality reduced by PCA. The input matrix with the response to the samples was prepared by assigning a number to every class in the input matrix of known samples.

A trained model was used to classify the data matrix of unknown samples (Data). A matrix of unknown samples (Data) was created from all samples that correspond to all stroke types, sizes, and positions according to the hypothesis tested. Different data were always used to train and test the algorithm to verify its generalization capabilities. Additional information for analysis (stroke size and position, phantom scale in x-axes and y-axes) contains an equivalent info matrix (DataInfo). Data from this matrix were used exclusively for the evaluation of problematic classifications.

The classification accuracy (CL-accuracy) and cross-validation error (CV-error) were calculated. A confusion matrix was used to determine which type of stroke was problematic for classification.

### 2.8. Hypothesis

**Hypothesis** **1** **(H1).** *The most suitable training data are from subjects with small strokes.*

Data corresponding to patients with small ischemic and hemorrhagic stroke sizes show the smallest deviation from data for healthy subjects. It can be expected that they represent the worst case for classification and therefore may be the most relevant for determining support vectors. We trained the SVM algorithm with datasets including successively only 20, then 30, and finally, 40 mm iStrokes and hStrokes placed at 20 positions in 10 randomly scaled phantoms (in total, 600 for each stroke type for testing). The SVM algorithm classified the remaining data (DataSet 1). Dimensionality reduction using PCA was not used in this case. For simplification, only data for 1 GHz were used. The data used for training and testing are described in Table 3.

**Hypothesis** **2** **(H2).** *Single-frequency and multi-frequency training data lead to the same classification accuracy.*

For a smaller number of frequency points, the algorithm works with a smaller amount of data. On the other hand, for a higher number of frequency points, we can reduce the dimensionality to only useful information. Classification results were compared for the SVM algorithm trained on data for a single frequency point (1 GHz), five frequency points (from 0.8 GHz, step 0.1 GHz, to 1.2 GHz), fifteen frequency points (from 0.8 GHz, 0.05 GHz step, up to 1.5 GHz) data and twenty-five frequency points (from 0.8 GHz, 0.05 GHz step, up to 2 GHz) data. We train the SVM algorithm for 20 mm iStroke and hStroke sizes located at 20 positions in 10 random scaled phantoms. The SVM algorithm classified the remaining data (DataSet 1). The data used for training and testing are described in Table 4. Complete graphs of classification accuracy and cross-validation error for different dimension reductions are provided in Appendix B.

**Hypothesis** **3** **(H3).** *SVM trained on data of subjects with small strokes can accurately classify randomly sized strokes*.

We trained the SVM algorithm using data on subjects with 20 mm iStroke and hStroke located at 20 positions in 10 randomly scaled phantoms (DataSet 1). We used training data for a frequency of 1 GHz and 90 dimensions reduced by PCA. The SVM algorithm classified the data of subjects with random stroke sizes (DataSet 2). The data used for training and testing are described in Table 5.

**Hypothesis** **4** **(H4).** *SVM trained on data of subjects with small strokes can accurately classify randomly sized strokes at random positions.*

We trained the SVM algorithm using data for 20 mm iStroke and hStroke sizes located at 20, 83, and 456 positions in 10 randomly scaled phantoms (DataSet 1). We used training data for a frequency of 1 GHz and 90 dimensions reduced by PCA. The SVM algorithm classified data for random stroke sizes and random stroke positions (DataSet 3). The data used for training and testing are described in Table 6. 

## 3. Results

### 3.1. Numerical Model Validation

**Figure 6:**
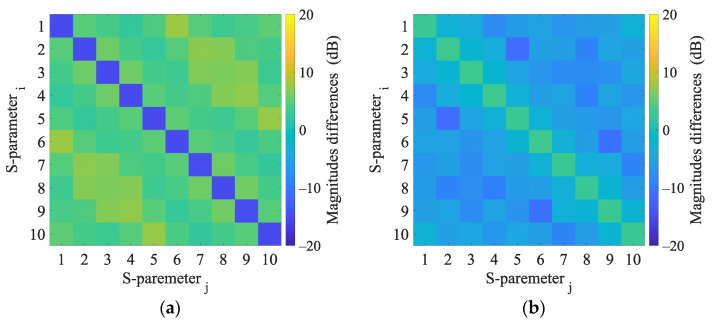
Magnitudes differences of 2D numerical model and 3D numerical model (**a**) and magnitudes differences of 3D numerical model and experimental measurement (**b**).

Numerical model validation was done by direct comparison of measured S-parameters from laboratory prototype of the microwave imaging system and S-parameters from 2D and 3D numerical simulations (see Figure 6).

Results of 2D and 3D numerical analysis of the changes in S-parameters induced by the virtual presence of the stroke model are listed in Appendix C. The reflection coefficients did not change when the stroke was inserted. Changes in the transmission coefficients were observed and were different for ischemic and hemorrhagic stroke and therefore applicable for the given purpose. Results in the 2D model cause more significant changes in the transmission coefficients than in the 3D model.

### 3.2. Principal Component Analysis

**Figure 7:**
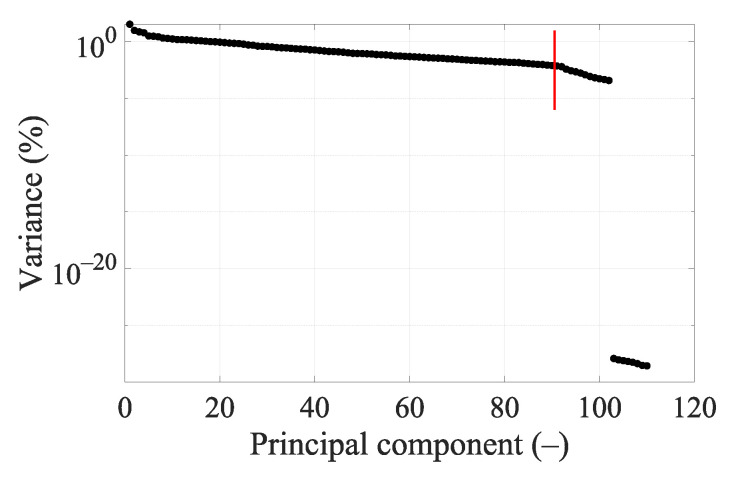
Principal components’ variance graph for DataSet 1 at 1 GHz. The red line indicates data reduction to 90 dimensions.

**Figure A2:**
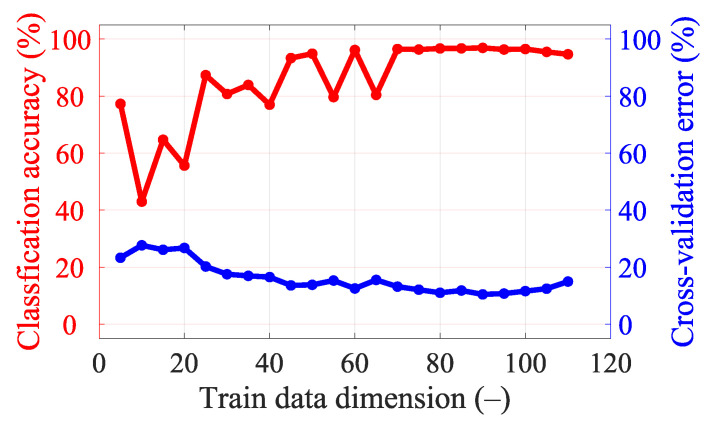
Classification accuracy and cross-validation error for different dimensionality reductions in data for a single frequency point (1 GHz).

The variance of DataSet 1 determined by principal component analysis (PCA) is shown in Figure 7. Around the 90th dimension, the variance begins to decrease more rapidly. Several higher principal components show significantly lower variance than others. Based on Figure 7 and Figure A2, where the classification accuracy was the highest and the cross-validation error the lowest, we decided to set 90 dimensions for a frequency of 1 GHz.

### 3.3. Hypothesis

**Hypothesis** **1** **(H1).** *The most suitable training data are from subjects with small strokes.*

SVMs were successively trained with data for different stroke sizes and then classified the remaining strokes (Training and testing data are described in Table 3). By training the algorithm with data corresponding to the smallest considered stroke sizes (with a diameter of 20 mm), the highest classification accuracy was achieved (see Table 7), even when classifying strokes up to the maximum considered diameter (40 mm). Thus, the hypothesis that the training data for small stroke sizes is the most suitable for training the SVM algorithm for stroke classification was confirmed.

**Hypothesis** **2** **(H2).** *Single-frequency and multi-frequency training data lead to the same classification accuracy.*

Graphs of classification accuracy and cross-validation error for different values of dimensionality and different numbers of frequency points are in Appendix B. From Table 8, we conclude that for SVM training, it turns out to be most appropriate to use 1 or 5 frequency points, where the classification accuracy and cross-validation error were almost identical. For 15 and 25 frequency points the results are significantly worse and only after dimensionality reduction using PCA do the results reach similar classification accuracy as with a lower number of frequency points. Thus, the hypothesis that the training data for one frequency point is sufficient for an accurate classification of stroke using the SVM algorithm was confirmed.

**Hypothesis** **3** **(H3).** *SVM trained on data of subjects with small strokes can accurately classify randomly sized strokes*.

**Figure 8:**
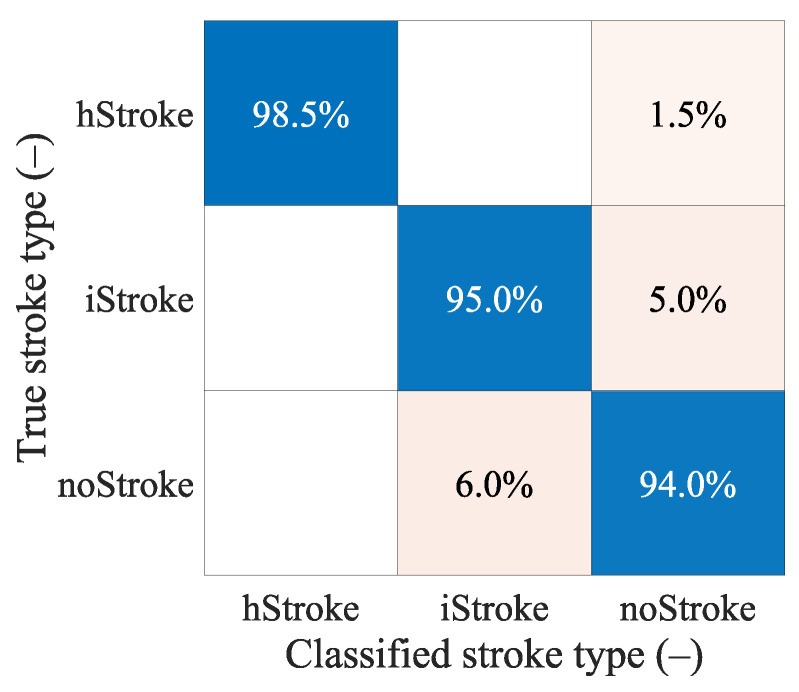
The confusion matrix for the classification of random ischemic and hemorrhagic stroke sizes.

The confusion matrix in Figure 8 shows that the classification between the iStroke and the hStroke class was accurate, only a 6.5% strokes were classified as the noStroke class, and 6.0% noStrokes were classified as the iStroke class. The hypothesis that the SVM algorithm for stroke classification trained on small strokes can classify random-size strokes was confirmed.

**Hypothesis** **4** **(H4).** *SVM trained on data of subjects with small strokes can accurately classify randomly sized strokes at random positions.*

**Figure 9:**
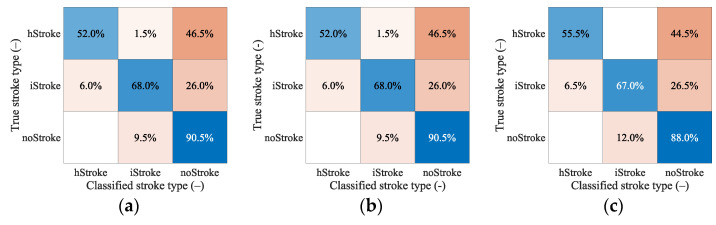
The confusion matrixes for classification of random stroke sizes and random stroke positions. In total, 200 samples for iStroke, 200 samples for hStroke and 200 samples for noStroke. SVM algorithm was trained for (**a**) 20 and (**b**) 83 ischemic and hemorrhagic strokes in 10 scaled phantoms and (**c**) for 456 ischemic and hemorrhagic strokes in 1 randomly selected phantom from 10 scaled phantoms.

From Table 9 we see that even for a larger amount of data, the algorithm cannot classify random data accurately and reach a maximum at around 70% classification accuracy. From the confusion matrixes in Figure 9, we can observe the correct and incorrect classification of iStroke, hStroke, and noStroke classes. The hypothesis that the SVM algorithm for stroke classification trained on small strokes can classify random size and random position strokes was not confirmed.

## 4. Discussion

The results of this work are for a large dataset for the training and testing of machine learning algorithms for MW detection and classification of cerebrovascular events. The dataset can be used to optimize the setting and study the performance and limits of the algorithms for the considered application. As part of this work, the aforementioned dataset was used to test selected hypotheses. The second result of this work is hypothesis testing, where it was proven that (a) the data of subjects with strokes of sizes and distributed throughout the brain are the most suitable for training SVM algorithms, (b) the SVM algorithm can reliably detect and classify iStrokes and hStrokes at a single frequency, and multi-frequency data do not bring an increase in classification accuracy, (c) a large amount of data of subjects with small strokes must be used to train the SVM algorithm. 

### 4.1. Comparison of S-Parameters Obtained by 2D and 3D Simulations and Measurements

In general, the 2D numerical model does not consider the propagation of EM waves in the 3rd dimension. In this work, a global optimization method was used to find the size of the boundary condition of the antenna port, which guarantees a good match between the measured and simulated transmission coefficients. Therefore, the magnitudes of the transmission parameters calculated and measured are in agreement; see Figure 6. On the other hand, the agreement in the reflection coefficients is worse. The calculated S-parameters are symmetric according to its main diagonal, while the measurements S-parameters show slight symmetry in the second decimal place in dB. These differences can be caused by noise or temperature fluctuations and must be considered when detecting a stroke.

### 4.2. S-Parameter Variability due to the Presence of Strokes

Changes in S-parameter values induced by the presence of ischemic and hemorrhagic stroke were studied using 2D and 3D numerical simulations. While the reflection coefficients did not change when the stroke was inserted, changes in the transmission coefficients were observed even for the worst-case scenario with the stroke located in the middle of the brain. The changes in transmission coefficients were different for ischemic and hemorrhagic stroke and therefore applicable for the given purpose. It is also possible to observe changes in the transmission parameters for the stroke location (–20, 30).

When comparing the calculated changes in the transmission coefficients in 2D and 3D, it is evident that not considering the EM wave propagation in the 3rd dimension causes more significant changes in the transmission coefficients than in the 3D model. Stroke detection and classification on 2D data can perform better than on real data or data obtained through 3D numerical simulations.

### 4.3. PCA

Based on the knowledge of the measured data and the information that the s-matrix from numerical simulations and measurements is symmetric, only the independent S-parameters of the matrix were selected, which resulted in the first reduction in the data dimension. Dimensionality reduction was tested using PCA for different frequency points.

**Figure A3:**
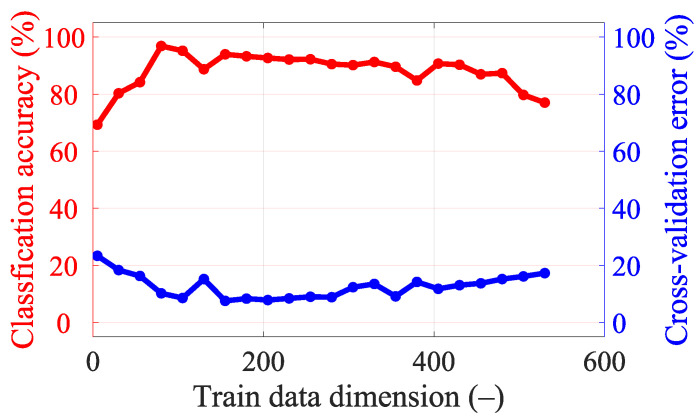
Classification accuracy and cross-validation error for different dimensionality reductions in the data for five frequency points (from 0.8 GHz, step 0.1 GHz, to 1.2 GHz).

**Figure A5:**
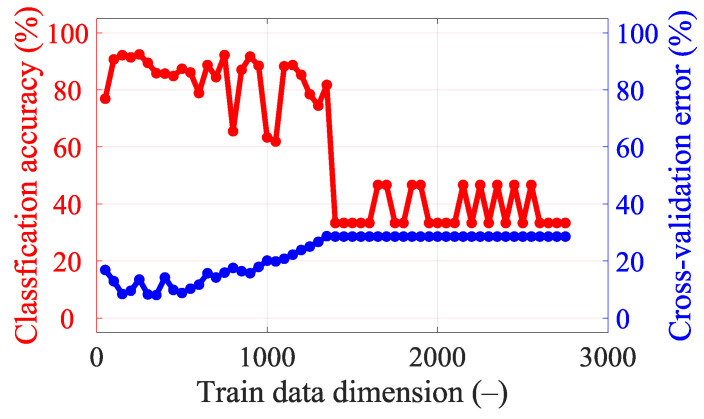
Classification accuracy and cross-validation error for different dimensionality reductions in the data for 25 frequency points (from 0.8 GHz, step 0.05 GHz, to 2 GHz).

With 25 frequency points, it is necessary to strongly reduce the data using PCA to achieve good classification accuracy results (see Figure A5), but we still did not achieve a higher classification accuracy or lower cross-validation error compared to a smaller number of frequency points (see Table 8). For five frequency points it is again necessary to strongly reduce the data using PCA to achieve good classification accuracy results (see Figure A3). The classification accuracy and cross-validation error for five frequency points are almost identical to one frequency point (see Figure A2), where only a small data reduction from 110 to 90 dimensions is needed to achieve the best classification accuracy results. This result is also suggested by the variance of the data (see Figure 7), where around 90 dimensions, the principal component starts to radically lose variance, and even a few principal components reach a significantly lower variance than most of the data. We decided to set 90 dimensions for extraction using PCA, where the data contain 99.9% variance.

### 4.4. Hypothesis H1

From Table 7, we can conclude that when training the algorithm only on the data of subjects with the smallest strokes (diameter 20 mm), the SVM algorithm achieved the highest classification accuracy of subjects with larger strokes up to a diameter of 40 mm. On the other hand, the cross-validation error increased from 5.5% to 11.8%. We assume that data of subjects with larger strokes contain higher variability; therefore, it is easier to classify them, and the algorithm achieves a smaller cross-validation error when training for larger strokes. Useful information for creating a support vector is provided by small-stroke subjects; however, they are more difficult to divide, which is why the Cross-validation error is higher than for larger strokes.

### 4.5. Hypothesis H2

SVM trained on data containing one and five frequency points showed almost identical classification accuracy and cross-validation error. They also outperformed an SVM trained with data containing a larger number of frequency points, even though the performance of the latter was enhanced by dimensionality reduction using PCA. This means that for this particular application, it would be enough to use a narrowband imaging system.

### 4.6. Hypothesis H3

The confusion matrixes in Figure 8 shows that the classification between ischemic and hemorrhagic stroke was accurate and only 6.5% of strokes were classified as non-stroke scenario and 6.0% of no strokes were classified as ischemic stroke.

Pre-hospital MW detection and classification of cerebrovascular events have the following specifics. Ischemic strokes need to be detected because they can be treated immediately with thrombolytic therapy. A situation where a hemorrhagic stroke is classified as ischemic is completely unacceptable. Thrombolytic therapy in case of hemorrhagic stroke causes hematoma enlargement and increases the risk of mortality [23]. In our study, unacceptable misclassification was observed only when we classify random stroke sizes and random stroke positions (DataSet 3), but the training dataset is extended by more stroke positions to eliminate misclassifications. It is, therefore, possible to consider only a 2-class classification into ischemic cerebrovascular events and the rest.

### 4.7. Hypothesis H4

The hypothesis that the SVM algorithm for stroke classification trained on data of the subjects with small strokes can accurately classify random size and random position strokes were not confirmed. From Table 9, we can conclude that only 20 iStroke and hStroke positions were not enough and the SVM algorithm only achieved a 63.5% classification accuracy. For 83 and 456 iStroke and hStroke positions SVM algorithm achieved only classification accuracy of 70% and 71%, respectively. Furthermore, we observed that the box constraint, which was determined by the optimization of the hyper-parameters, reaches high values. High box constraint values suggest good separation and fewer misclassifications, but unknown data for random stroke size and random stroke positions, the SVM algorithm is not able to classify correctly, and achieves only 70% classification accuracy. We also checked which samples were misclassified, but no pattern was discovered. From the confusion matrixes in Figure 9 we conclude that most of the inaccurate classifications belonged to the category without stroke, and these stroke patients will be taken to the hospital anyway. The biggest problem in the treatment of stroke patients is the classification of hemorrhagic strokes into the ischemic category. The use of anticoagulation in hemorrhagic stroke causes hematoma enlargement and increases the risk of mortality [23]. For this situation, there were no misclassifications for the 456 iStroke and hStroke positions used for SVM algorithm training.

### 4.8. Comparison with Published Studies

The results are partially comparable to [10], where a study of the feasibility of subdural hematoma classification by the SVD-based algorithm was performed on synthetic data from 2D numerical simulations. The authors achieved 82–96% classification success rate, considering only a two-class classification of healthy subjects and subjects with subdural hematomas. Ischemic stroke cases were not considered. Subdural hematoma is distinct from intracerebral hemorrhage, which is the most common type of hemorrhage in stroke patients. Intracerebral hemorrhage is located within the brain tissue and is usually of spherical shape and is generally characterized by a smaller volume than subdural hematoma; hence, the subdural hematoma may be classified with lower error. The 2D model is a significant simplification; therefore, a 3D numerical study was presented in [9]. Again, a two-class classification is performed. In our study, the results of classification into three classes are presented.

In [24], an alternative and efficient method was proposed to create the training dataset, based on the distorted Born approximation, to obtain a linear scattering operator from the dielectric contrast space to the scattering parameters’ one. A dataset containing 10,000 samples was created in a relatively short time and with low computational effort. On the other hand, a single 3D CAD model of a human head was used and scaled to obtain a total of 10 head models. In our study, a higher data variability was considered because all 10 different head models were scaled randomly in each numerical simulation.

The algorithms were tested in [24] on a large amount of data obtained from the linear dispersion operator. An evaluation of the suitability of different datasets for training was not carried out. In our study, the effect of multi-frequency data on classification accuracy was tested, but the results show and agree with the statement in [24] that single-frequency data is sufficient for this application.

Classification using the SVM algorithm with a different data processing approach was performed in [7]. Data were obtained for 10 head models without scaling and only for ellipsoid strokes of 10 mm^3^ to 35 mm^3^. A total of 100 stroke head models were created. A two-class classification was performed between iStroke and hStroke classes. NoStroke class was not included. Inverse FFT was used to convert these signals to time series signals. Data variability was increased by adding noise, which also significantly reduced the classification success rate from 94% to 77%. In [8], dimensionality reductions by PCA led to classification success rate increase to 99%. The increase in the classification success using PCA also confirms our results, even though it was a different data processing approach.

### 4.9. Further Plans

Training data for machine learning algorithms should ideally be obtained from measurements performed in subjects with acute strokes. However, these patients are in a life-threatening situations. Using 2D numerical simulations, we obtained the large amount of data needed to train and test machine learning algorithms and to optimize their settings. We can test various hypotheses to understand well the performance and limits of this application. In future, we plan to perform 3D numerical simulations for a larger number of human heads and a more realistic MW imaging system with up to 24 antennas. Algorithms trained on the data obtained from numerical simulations would probably fail in the real world. Therefore, we plan to test the algorithm on experimentally obtained measurement data using a MW system [25] and anatomically and dielectrically realistic phantoms of the human head such as those proposed in [26]. Machine learning algorithms do not provide the position or magnitude of the stroke in the human head; therefore, in the future, we propose to combine the SVM algorithm with the TSVD Born approximation to obtain an image of the observed area. Both algorithms can be used on the same device.

## 5. Conclusions

Machine learning algorithms appear to be a promising method for MW stroke detection and classification. A large dataset for the training and testing of machine learning algorithms for MW detection and classification of cerebrovascular events was created. We demonstrated that the SVM algorithm is able to detect the presence of the stroke and classify it as ischemic and hemorrhagic classes. Data from a single frequency point (1 GHz) are sufficient for training the accurate SVM predictors. Further, it was shown that the data of subjects with smaller strokes appear to be the most suitable for training accurate SVM predictors with high generalization capabilities for stroke-trained position placement. The study indicate that it is difficult to find suitable training data to accurately detect and classify the type of stroke located at an arbitrary position in the head.

## Figures and Tables

**Table 1:** Dielectric properties of the domain in the human head modes at 1 GHz [21].

Tissue Layer	*ε_r_* (−)	σ (S/m)
Scalp ^†^	35.68	0.66
Skull	12.34	0.16
CSF	68.44	2.46
Brain ^‡^	40.00	1.00
Ischemic stroke	34.00	0.85
Hemorrhagic stroke	61.07	1.58
Matching Medium *	40.00	1.00

^†^ Average parameters of skin and fat. ^‡^ Average parameters of grey matter, white matter, and cerebellum. * Matching medium dielectric properties are given by the IEEE standard [22].

**Table 2:** Model parameters in the DataSets.

DataSet (−)	Stroke Type (−)	Stroke Sizes (mm)	Stroke Positions (−)	Head Phantoms (−)	Head Scaled (%)	Frequency Points (−)
1	hStroke iStroke noStroke	20, 25, 30, 35, 40	Fixed 20 + 83 + 456	10	95–105 ^†^	25
2	hStroke iStroke noStroke	20–40 ^†^	Fixed 20	10	95–105 ^†^	25
3	hStroke iStroke noStroke	20–40 ^†^	Random 20 ^†^	10	95–105 ^†^	25

^†^ Uniform probability density random number generator.

**Table 3:** Training and testing data parameters for hypothesis H1.

	TrainData	TestData
DataSet (−)	No. 1	No.1
Stroke type (−)	hStroke, iStroke, noStroke	hStroke, iStroke, noStroke
Stroke sizes (mm)	20 or 30 or 40	20, 25, 30, 35, 40
Stroke positions (−)	Fixed 20	Fixed 20
Head phantoms (−)	10	10
Frequency points (−)	1	1
DataSet size ^†^ (−)	600	3000
DataSet dimensions ^‡^ (−)	110	110

^†^ The single measured S-matrices, ^‡^ The number of S-parameters from S-matrix.

**Table 4:** Training and testing data parameters for hypothesis H2.

	TrainData	TestData
DataSet (−)	No. 1	No. 1
Stroke type (−)	hStroke, iStroke, noStroke	hStroke, iStroke, noStroke
Stroke sizes (mm)	20	20, 25, 30, 35, 40
Stroke positions (−)	Fixed 20	Fixed 20
Head phantoms (−)	10	10
Frequency points (−)	1 or 5 or 15 or 25	1
DataSet size ^†^ (−)	600	3000
DataSet dimensions ^‡^ (−)	110 or 550 or 1650 or 2750	110 or 550 or 1650 or 2750

^†^ The single measured S-matrices, ^‡^ The number of S-parameters from S-matrix.

**Table 5:** Training and testing data parameters for hypothesis H3.

	TrainData	TestData
DataSet (−)	No. 1	No. 2
Stroke type (−)	hStroke, iStroke, noStroke	hStroke, iStroke, noStroke
Stroke sizes (mm)	20	20–40
Stroke positions (−)	Fixed 20	Fixed 20
Head phantoms (−)	10	10
Frequency points (−)	1	1
DataSet size ^†^ (−)	600	600
DataSet dimensions ^‡^ (−)	90	90

^†^ The single measured S-matrices, ^‡^ The number of S-parameters from S-matrix.

**Table 6:** Training and testing data parameters for hypothesis H4.

	TrainData	TestData
DataSet (−)	No.1	No.3
Stroke type (−)	hStroke, iStroke, noStroke	hStroke, iStroke, noStroke
Stroke sizes (mm)	20	20–40
Stroke positions (−)	Fixed 20 or 83 or 456	Random 20
Head phantoms (−)	10	10
Frequency points (−)	1	1
DataSet size ^†^ (−)	600 or 2490 or 1368	600
DataSet dimensions ^‡^ (−)	90	90

^†^ The single measured S-matrices, ^‡^ The number of S-parameters from S-matrix.

**Table 7:** Effect of stroke sizes on classification accuracy for DataSet1 at 1 GHz.

Stroke Size (mm)	CV-Error (%)	CL-Accuracy (%)
20	11.8	95.7
30	7.0	85.3
40	5.5	65.5

**Table 8:** The effect of number of frequency points and dimension reduction on the classification accuracy.

Frequency Points (−)	Frequencies (GHz)	Total Dimensions (−)	CL-Accuracy CV-Error (%)	Reduced Dimensions (−)	CL-Accuracy CV-Error (%)
1	1.00	110	94.6 14.9	90	96.9 10.4
5	0.80 1.00 1.20 1.40 1.50	550	77.0 17.3	80	96.8 10.2
15	0.80–1.50 step 0.05	1650	87.2 21.1	70	96.3 11.1
25	0.80–2.00 step 0.05	2750	33.3 28.6	150	92.2 8.4

**Table 9:** The SVM algorithm classification of random stroke sizes and random stroke positions with different amounts of training data (different number of 20 mm stroke positions in the head).

Training Data Stroke Positions (−)	Training Data Number of Phantoms (−)	CV-Error (%)	CL-Accuracy (%)	Hyperparameters Optimalization Settings (−)
20 hStroke 20 iStroke	10	11.3	64.3	Box Constraint = 912.92 Kernel Function = Gaussian Kernel Scale = 17.8
83 hStroke 83 iStroke	10	8.9	70.5	Box Constraint = 212.97 Kernel Function = Gaussian Kernel Scale = 11.41
456 hStroke 456 iStroke	1 randomly selected from 10 phantoms	11.3	70.2	Box Constraint = 385.03 Kernel Function = Gaussian Kernel Scale = 19.74

**Figure A1:**
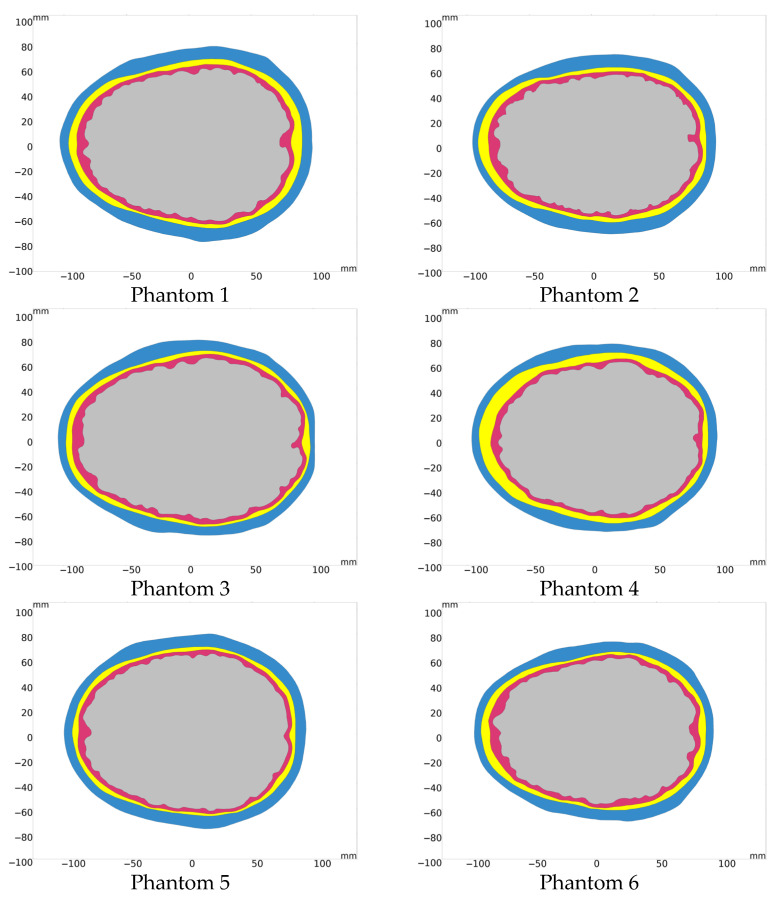

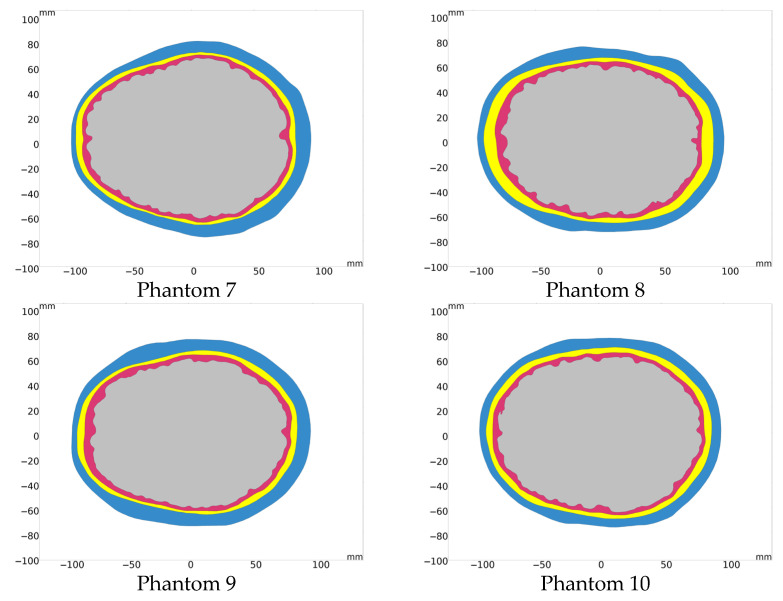
10 different human head model geometries based on real subjects.

**Figure A4:**
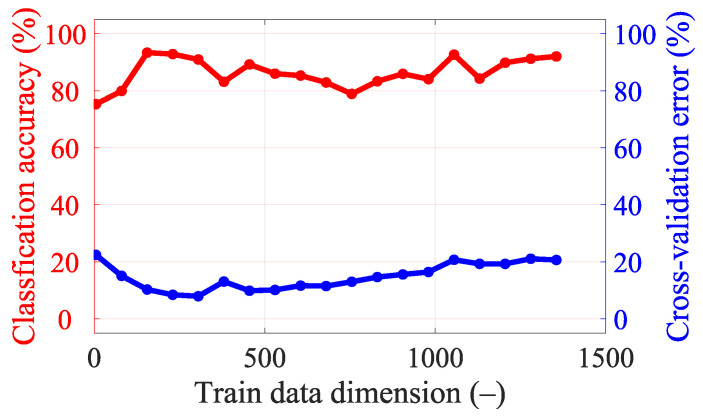
Classification accuracy and cross-validation error for different dimensionality reductions in data for 15 frequency points (from 0.8 GHz, step 0.05 GHz, to 1.5 GHz).

**Figure A6:**
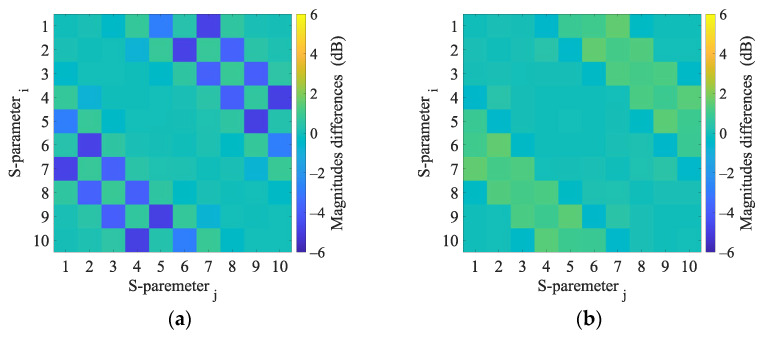
2D numerical simulations. Magnitudes difference of 2D numerical model induced by the virtual presence of a 4 cm radius (**a**) hemorrhagic and (**b**) ischemic stroke located at position (0, 0).

**Figure A7:**
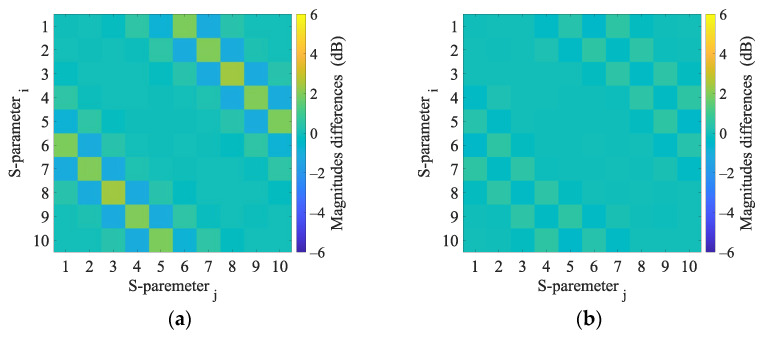
3D numerical simulations. Magnitudes difference of 3D numerical model induced by the virtual presence of a 4 cm radius (**a**) hemorrhagic and (**b**) ischemic stroke located at position (0, 0).

**Figure A8:**
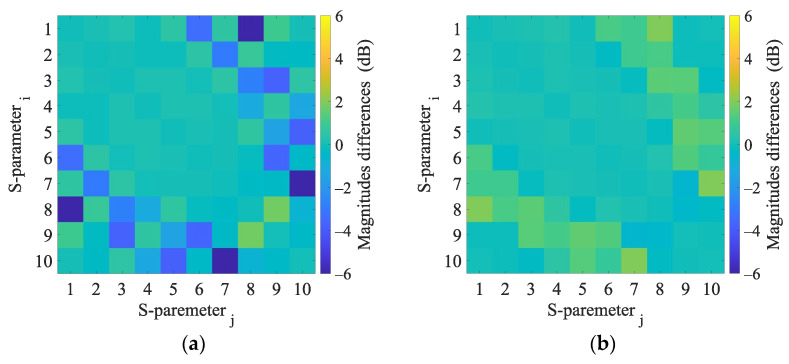
2D numerical simulations. Magnitudes difference of 2D numerical model induced by the virtual presence of a 4 cm radius (**a**) hemorrhagic and (**b**) ischemic stroke located at position (−20, 30).

**Figure A9:**
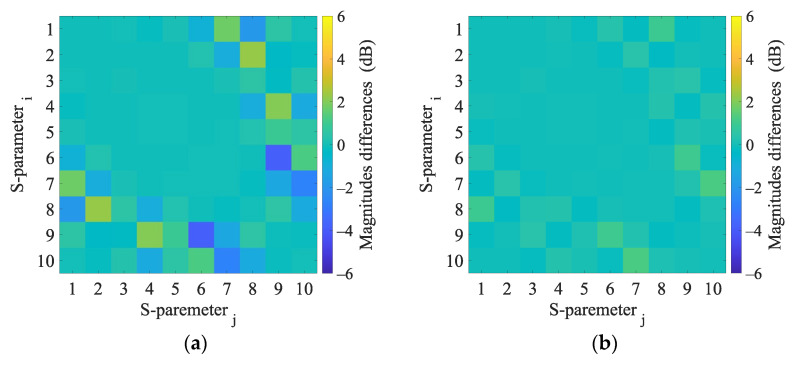
3D numerical simulations. Calculated differences in S-parameters induced by the virtual presence of a 4 cm radius (**a**) hemorrhagic and (**b**) ischemic stroke located at position (−20, 30).

## Data Availability

DataSets from 2D numerical simulations can be downloaded after filling out the form: https://forms.gle/K2WPhfsXgTmgJCnh6 (accessed on 9 February 2023).

## Appendix B

Graphs of classification accuracy and cross-validation error for different dimensionality reductions for different numbers of frequency points.

## Appendix C

This appendix presents the S-parameter changes induced by the virtual presence of ischemic and hemorrhagic strokes calculated using 2D and 3D numerical models. These changes were calculated by subtracting the S-parameters for the case when the inner part of the antenna system was filled only with the matching medium (εr = 40, σ = 1 S/m) from the S-parameters for the case where the stroke model was added virtually.
